# Continued Follow-Up of Phambili Phase 2b Randomized HIV-1 Vaccine Trial Participants Supports Increased HIV-1 Acquisition among Vaccinated Men

**DOI:** 10.1371/journal.pone.0137666

**Published:** 2015-09-14

**Authors:** Zoe Moodie, Barbara Metch, Linda-Gail Bekker, Gavin Churchyard, Maphoshane Nchabeleng, Koleka Mlisana, Fatima Laher, Surita Roux, Kathryn Mngadi, Craig Innes, Matsontso Mathebula, Mary Allen, Carter Bentley, Peter B. Gilbert, Michael Robertson, James Kublin, Lawrence Corey, Glenda E. Gray

**Affiliations:** 1 Vaccine and Infectious Disease Division, Fred Hutchinson Cancer Research Center, Seattle, Washington, United States of America; 2 Desmond Tutu HIV Foundation, University of Cape Town, Cape Town, South Africa; 3 Aurum Institute for Health Research, Johannesburg, South Africa; 4 School of Public Health, University of Witwatersrand, Johannesburg, South Africa; 5 London School of Hygiene and Tropical Medicine, London, United Kingdom; 6 Mecru Clinical Research Unit, Sefako Makgatho Health Sciences University, Pretoria, South Africa; 7 Centre for the AIDS Programme of Research in South Africa (CAPRISA), Durban, South Africa; 8 School of Laboratory Medicine and Medical Sciences, University of KwaZulu-Natal and National Health Laboratory Service, Durban, South Africa; 9 Perinatal HIV Research Unit, Faculty of Health Sciences, University of Witwatersrand, Johannesburg, South Africa; 10 Aurum Institute Clinical Research Site, Klerksdorp, South Africa; 11 Vaccine Research Program, Division of AIDS, NIAID, NIH, Rockville, United States of America; 12 Merck Research Laboratories, West Point, Pennsylvania, United States of America; 13 South African Medical Research Council, Cape Town, South Africa; Rush University, UNITED STATES

## Abstract

**Background:**

The Phase 2b double-blinded, randomized Phambili/HVTN 503 trial evaluated safety and efficacy of the MRK Ad5 *gag*/*pol*/*nef* subtype B HIV-1 preventive vaccine vs placebo in sexually active HIV-1 seronegative participants in South Africa. Enrollment and vaccinations stopped and participants were unblinded but continued follow-up when the Step study evaluating the same vaccine in the Americas, Caribbean, and Australia was unblinded for non-efficacy. Final Phambili analyses found more HIV-1 infections amongst vaccine than placebo recipients, impelling the HVTN 503-S recall study.

**Methods:**

HVTN 503-S sought to enroll all 695 HIV-1 uninfected Phambili participants, provide HIV testing, risk reduction counseling, physical examination, risk behavior assessment and treatment assignment recall. After adding HVTN 503-S data, HIV-1 infection hazard ratios (HR vaccine vs. placebo) were estimated by Cox models.

**Results:**

Of the 695 eligible, 465 (67%) enrolled with 230 from the vaccine group and 235 from the placebo group. 38% of the 184 Phambili dropouts were enrolled. Enrollment did not differ by treatment group, gender, or baseline HSV-2. With the additional 1286 person years of 503-S follow-up, the estimated HR over Phambili and HVTN 503-S follow-up was 1.52 (95% CI 1.08–2.15, p = 0.02, 82 vaccine/54 placebo infections). The HR was significant for men (HR = 2.75, 95% CI 1.49, 5.06, p = 0.001) but not for women (HR = 1.12, 95% CI 0.73, 1.72, p = 0.62).

**Conclusion:**

The additional follow-up from HVTN 503-S supported the Phambili finding of increased HIV-1 acquisition among vaccinated men and strengthened the evidence of lack of vaccine effect among women.

**Trial Registration:**

clinicaltrials.gov NCT00413725

SA National Health Research Database DOH-27-0207-1539

## Introduction

Developing a safe and effective vaccine against HIV-1 acquisition remains a public health priority and a scientific challenge three decades after the discovery of the HIV-1 virus and the launch of the first HIV-1 vaccine clinical trial. Of the six large-scale HIV-1 vaccine efficacy trials conducted in humans to date, only the RV144 Thai trial testing a canarypox vectored prime (ALVAC-HIV)/ALVAC + gp120 boost vaccine regimen has shown a modest prevention effect (31%) [1]. Two earlier trials of a recombinant bivalent gp120 vaccine, one in a Thai injecting drug cohort and the other in a cohort primarily comprising men who have sex with men (MSM) in the Americas and the Netherlands, and a recent trial with a DNA prime recombinant adenovirus serotype-5 (rAd5) boost vaccine regimen among MSM in the United States showed no efficacy [2–4]. Two other trials [5, 6] of a rAd5-vectored vaccine expressing HIV-1 internal proteins Gag, Pol and Nef also showed no vaccine efficacy and raised the possibility of increased HIV-1 acquisition in the vaccine group despite clinical safety and immunogenicity demonstrated in multiple phase1/2 studies [7–15].

The Step study tested the MRK rAd5 vectored polyvalent HIV-1 *gag/pol/nef* subtype B vaccine in a cohort consisting primarily of MSM and at-risk women in the Americas, Caribbean and Australia, where the circulating HIV-1 is of subtype B. In September 2007, the first interim efficacy analysis concluded that the trial met the pre-specified criteria for futility, thus vaccinations were halted, follow-up continued, and participants were informed of their treatment assignments. Subgroup analyses among Ad5 seropositive and/or uncircumcised men at that time showed an increased susceptibility to HIV-1 acquisition in those who received vaccine vs. placebo (based on interaction tests) [5]; analyses by Ad5 serostatus subgroups were pre-specified however the analyses of Ad5 status and circumcision subgroups were not. At the end of four years of follow-up, for all participants the vaccine:placebo hazard ratio [HR] for HIV-1 infection was 1.40 (95% CI 1.03–1.92; p = 0.03) [16]. The vaccine-induced enhanced risk of HIV-1 acquisition seen in the Ad5 seropositive and/or uncircumcised men was more evident in the first 18 months of follow-up and appeared to wane over time, whereas for circumcised, Ad5 seronegative men, the vaccine:placebo HR increased over time from 0.38 (95% CI 0.16–0.90) in the first 18 months to 2.18 (95% CI (0.97–4.92) after 18 months of follow-up. There were too few infections among women to assess vaccine efficacy.

The second Phase 2b trial of the MRK rAd5 vaccine, HVTN 503 or Phambili [6, 17], was undertaken in South Africa, where clade C is the predominant HIV-1 subtype, in a principally heterosexual population. Enrollment and vaccinations were halted in September 2007 when the Step study met the non-efficacy criteria. By that time, Phambili had enrolled 800 HIV non-infected participants aged 18–35 years (median 22), 55% of whom were men. Participants were unblinded to their treatment assignments and followed for 42 months. The initial Phambili analysis based on a median of 25 months of follow-up reported a higher number of HIV-1 infections in the vaccine vs placebo groups but no significant difference overall or by gender [17]. Over the entire Phambili follow-up period (with median follow-up of 42 months), a significantly higher rate of HIV-1 infections was seen in the vaccine group than in the placebo group, with the difference stronger for men than for women [6]. This difference could not be explained by number of vaccinations received, dropout or self-reported risk behaviors.

Although there was no difference in retention between vaccine and placebo groups over the 42 months of follow-up in Phambili, the early trial unblinding of participants and staff and the potential of imbalanced undetected HIV-1 infections among participants who dropped-out may have affected results. To better assess the HIV acquisition risk, the study investigators undertook a follow-up study, HVTN 503-S, to recall all HIV-1 uninfected participants for an additional HIV test. This report describes the success of this recall effort and provides estimates of the vaccine effect on HIV-1 acquisition that include HIV infection data from the HVTN 503-S recall study.

## Methods

### Study Design

HVTN 503-S was designed to enroll the 695 Phambili participants who were HIV uninfected and known to be alive at the end of their Phambili follow-up for additional HIV testing. Participants were from all 5 South African Phambili sites (Soweto, Cape Town, Klerksdorp-Orkney-Stilfontein-Hartbeestfontein [KOSH], eThekwini, and MEDUNSA). The primary objectives were to expand HIV diagnostic testing coverage and to expand information on behavioral risk. After providing informed consent in which participants were informed about the Phambili study results, participants received a physical examination, which for men included circumcision status assessment; received HIV counseling and testing; received HIV risk reduction counseling; and were assessed for their treatment assignment recall. Staff also administered a short behavioral risk assessment that asked about change in sexual practices (frequency of sex, number of partners, and condom use) between the participant’s last year in the Phambili trial compared to the following year; response categories were no change, increased or decreased. Participants who tested HIV positive had an additional visit to confirm the result. A concern was that male circumcision status may not have been adequately assessed on Phambili since 39% of reports of circumcision at enrollment were based on self-report and complete and partial circumcisions were not distinguished. For HVTN 503-S, the physical examination required observation of circumcision status unless the participant declined, and staff were specifically trained to distinguish partial from complete circumcisions.

Because vaccine recipients may have had vaccine-induced seropositivity, HIV testing for vaccinees was performed by the National Institute for Communicable Diseases (NICD, Johannesburg, South Africa) following the testing algorithm used in Phambili. For placebo recipients, the site or local lab performed testing following their site guidelines and referred additional specimens to NICD for RNA PCR testing for any participants who had a possible recent HIV-1 exposure or evidence of acute infection.

Each site had a six month enrollment window from protocol activation. Sites used various methods to contact Phambili participants which included text messaging and phone calls to the participant’s last known phone number; home visits by field workers, Community Advisory Board (CAB) and study retention officers; advertising in local newspapers and on radio; and use of loud speakers from vehicles driving in areas where Phambili participants were initially recruited.

The study was registered with the Food and Drug Administration in the USA as an amendment to the Phambili investigational new drug application and was approved by the ethical review committees and the institutional review boards of the University of the Witwatersrand, University of Cape Town, University of Limpopo and the University of Kwazulu-Natal. Participants provided written informed consent in English or their local language.

### Statistical Analysis

All analyses were modified intention-to-treat (MITT). The MITT population included all vaccinated participants apart from those diagnosed as infected with HIV-1 on the day of first vaccination. Differences between categorical variables were tested with Chi-square tests or Fisher’s exact tests for 2 X 2 tables. Multivariable logistic regression models were used to assess predictors of enrollment in HVTN 503-S, including Phambili treatment group, gender, age, HSV-2 serostatus at enrollment in Phambili, circumcision status at the end of Phambili follow-up (men), early discontinuation of Phambili follow-up and HIV risk behaviors reported at their last Phambili visit (multiple partners, unprotected sex, drinking or drug use with sex, no main partner, heavy drinking or dagga [marijuana] use). Kaplan-Meier cumulative incidence plots of time to HIV-1 infection by treatment groups for the combined Phambili and HVTN 503-S follow-up times are provided for the entire cohort and by gender subgroups, with log-rank tests used to determine statistical significance. Cox proportional hazards models were used to estimate the HR for HIV-1 infection due to vaccination (vaccine:placebo), overall and within subgroups. Time-dependent Cox models were used when circumcision was included in the model as many men were circumcised while on study. Differences in HRs between subgroups were assessed with Wald tests for the vaccine by subgroup interaction. Time to HIV-1 infection for participants infected while on Phambili was defined as the time from first Phambili study injection to the midpoint between the last plasma HIV-1 RNA negative and first RNA positive test; for HIV-1 infections diagnosed at the HVTN 503-S visit, the midpoint was determined from the time of the last plasma HIV-1 RNA negative test in Phambili. For uninfected participants, the censoring time was defined as the time from first study injection to the HVTN 503-S HIV test or last Phambili test for those not enrolled in HVTN 503-S. Circumcision status was based on the HVTN 503-S physical assessment; partial circumcisions were considered as uncircumcised in the analysis. For men refusing the HVTN 503-S examination or who were not enrolled in HVTN 503-S, their Phambili circumcision assessment was used. Results presented for the Phambili follow-up period include the additional follow-up and assessment data for all HVTN 503-S participants and updated baseline HSV-2 data for 2 participants. All p-values are 2-sided. SAS and R were used to run all analyses.

## Results

HVTN 503-S began enrolling participants in June 2013 and follow-up was completed in January 2014. Of the 695 participants targeted for enrollment in HVTN 503-S, 57.3% were men, 48.2% had received vaccine, and 26.5% had discontinued follow-up on Phambili early. 465 (66.9%) of the 695 were enrolled, with a significant difference in enrollment percentages between study sites (p<0.001) and by circumcision status at the end of Phambili follow-up (p = 0.045), but not by Phambili treatment groups, gender, Ad5 serostatus, age, or by baseline HSV-2 status (table 1). Enrollment rates were higher for those who completed Phambili follow-up than those who discontinued early overall and within gender subgroups (p-values < 0.001). Among the 184 participants who discontinued Phambili follow-up early, 70 (38.0%) were enrolled in HVTN 503-S, with no significant difference by treatment group. More vaccinees were enrolled than placebo recipients at Soweto (p = 0.045) and Cape Town (p = 0.03); no significant differences were seen in enrollment rates at other sites. Multivariable logistic regression models identified study site (p<0.001) and discontinuation of Phambili follow-up (p<0.001) as predictors of enrollment, but not demographics or HIV risk behaviors. Reasons for non-enrollment (n = 230) were: 97 unable to be contacted, 77 relocated away from a study site, 36 unable to be scheduled for a visit or no show for their scheduled visit, 15 not interested, 3 died from unknown causes after their last Phambili visit, and for 2 the enrollment period ended prior to making contact (S1 Fig). There were no differences in reasons by treatment arm or gender (data not shown). The median time between last Phambili and the HVTN 503-S visit was 33 months (IQR 31, 35). The extended follow-up added 1,286 person-years of follow-up to the 2,268 person-years observed during Phambili.

**Table 1:** HVTN 503-S contact disposition for eligible Phambili participants (N = 695).

	All		Vaccine^d^	Placebo^d^
Characteristic	# Enrolled/ # eligible (%)	p-value for subgroup comparison	# Enrolled/# eligible (%)	# Enrolled/# eligible (%)
Overall	465/695 (66.9%)		230/335 (68.7%)	235/360 (65.3%)
**Sex**		0.25		
Men	259/398 (65.1%)		130/193 (67.4%)	129/205 (62.9%)
Women	206/297 (69.4%)		100/142 (70.4%)	106/155 (68.4%)
**Circumcision status at end of Phambili follow-up** ^a^		0.045		
Circumcised men	176/252 (69.8%)		94/124 (75.8%)	82/128 (64.1%)
Uncircumcised men	83/139 (59.7%)		36/66 (54.6%)	47/73 (64.4%)
**Ad5 serostatus**		0.36		
Ad5 seropositive	370/560 (66.1%)		187/271 (69.0%)	183/289 (63.3%)
Ad5 seronegative	95/135 (70.4%)		43/64 (67.2%)	52/71 (73.2%)
**Age**		1.0		
Age ≤ 25 years	335/501 (66.9%)		163/243 (67.1%)	172/258 (66.7%)
Age > 25 years	130/194 (67.0%)		67/92 (72.8%)	63/102 (61.8%)
**Study site**		<0.001		
Soweto	223/266 (83.8%)		111/125 (88.8%)	112/141 (79.4%)
Cape Town	75/139 (54.0%)		43/67 (64.2%)	32/72 (44.4%)
KOSH	106/204 (52.0%)		48/102 (47.1%)	58/102 (56.9%)
eTwekwini	25/40 (62.5%)		10/18 (55.6%)	15/22 (68.2%)
Medunsa	36/46 (78.3%)		18/23 (78.3%)	18/23 (78.3%)
**Phambili follow-up status**		<0.001 ^b^		
Completed	395/511 (77.3%)		194/249 (77.9%)	201/262 (76.7%)
Discontinued early	70/184 (38.0%)		36/86 (41.9%)	34/98 (34.7%)
**Baseline HSV-2 status** ^c^		0.13		
Seronegative	338/492 (68.7%)		165/235 (70.2%)	173/257 (67.3%)
Seropositive	126/201 (62.7%)		64/99 (64.6%)	62/102 (60.8%)

^a^ 7 men eligible for HVTN 503-S did not have a circumcision assessment after enrollment in Phambili.

^b^Differences in enrollment between those who completed Phambili follow-up and those who discontinued early were also statistically significant within gender subgroups (all p-values < 0.001).

^c^ Two participants were missing HSV-2 data at enrollment in Phambili.

^d^ Differences in enrollment between vaccine and placebo groups overall or within subgroups were not statistically significant, except for Soweto (p = 0.045) and Cape Town (p = 0.03); the p-value for circumcised men was 0.054.

**Fig 1:**
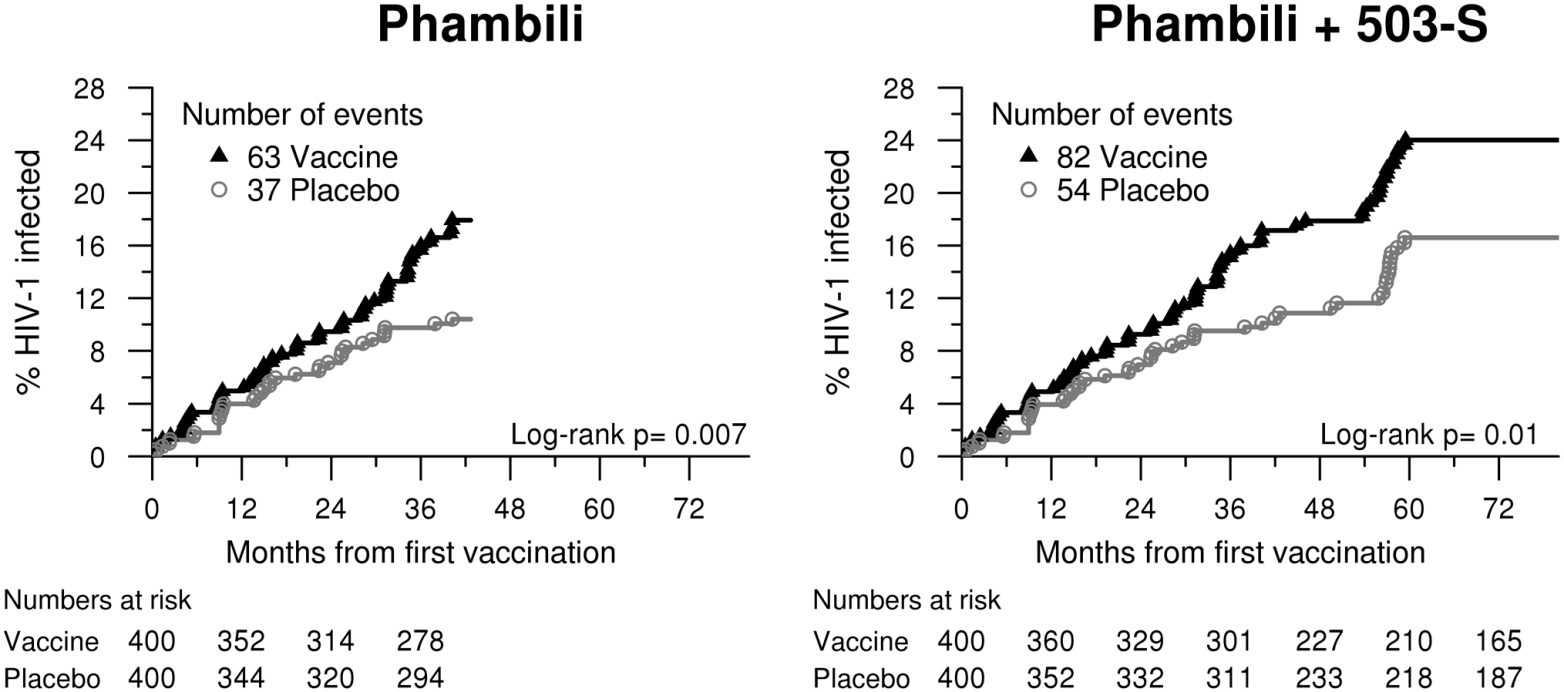
Cumulative HIV-1 incidence curves for vaccine and placebo groups for Phambili and Phambili + HVTN 503-S follow-up; Phambili + HVTN 503-S curves are based on all MITT Phambili participants (n = 800) with updated HIV-1 status for the subset of participants enrolled in HVTN 503-S (n = 465).

HVTN 503-S HIV testing identified 36 new HIV-1 infections, 19 in the vaccine group and 17 in the placebo group (table 2). In the combined Phambili and HVTN 503-S follow-up periods, significantly more HIV-1 infections were detected amongst vaccinees (n = 82) as compared to placebo (n = 54); the annualized HIV-1 incidence rate was 4.7% (95% CI 3.7%-5.8%) for vaccinees and 3.0% (95% CI 2.3%-3.9%) for placebo recipients (p = 0.01, Fig 1, table 2). The vaccine:placebo HR adjusted for HSV-2 status at the time of enrollment in Phambili was 1.52 (95% CI: 1.08, 2.15) in the combined Phambili and HVTN 503-S data, compared to 1.70 (95% CI: 1.13–2.55, p = 0.01) in Phambili alone(table 3); baseline HSV-2 status was a significant predictor of HIV infection thus the Cox models always adjust for this variable.

**Table 2:** Incident HIV-1 infections detected during Phambili and Phambili + HVTN 503-S follow-up periods.

	Overall	Vaccine				Placebo			
	Person-years	N	# infections	Annualized HIV-1 incidence rate	(95% CI)	N	# infections	Annualized HIV-1 incidence rate	(95% CI)
**Phambili follow-up**									
All	2269	400	63	5.6%	(4.3, 7.2)	400	37	3.2%	(2.3, 4.5)
Men	1269	222	28	4.4%	(2.9, 6.4)	219	11	1.7%	(0.9, 3.1)
Women	1000	178	35	7.2%	(5.0, 10.0)	181	26	5.1%	(3.3, 7.4)
**Phambili + 503-S follow-up**									
All	3555	400	82	4.7%	(3.7, 5.8)	400	54	3.0%	(2.3, 3.9)
Men	1998	222	39	3.9%	(2.8, 5.3)	219	14	1.4%	(0.8, 2.3)
Women	1557	178	43	5.6%	(4.1, 7.6)	181	40	5.0%	(3.6, 6.9)
Completed Phambili FU	2968	312	75	5.1%	(4.0, 6.4)	299	50	3.4%	(2.5, 4.4)
Discontinued Phambili FU	586	88	7	2.5%	(1.0, 5.1)	101	4	1.3%	(0.4, 3.4)
Men completed Phambili FU	1605	167	33	4.0%	(2.8, 5.7)	150	14	1.8%	(1.0, 3.0)
Women completed Phambili FU	1363	145	42	6.4%	(4.6, 8.6)	149	36	5.1%	(3.6, 7.1)
Men discontinued Phambili FU	393	55	6	3.4%	(1.2, 7.3)	69	0	0.0%	0.0, 1.7)
Women discontinued Phambili FU	193	33	1	1.0%	(0.0, 5.4)	32	4	4.5%	(1.2,11.4)

CI = confidence interval. HIV-1 = human immunodeficiency virus 1.

**Table 3:** Risk of HIV-1 infection (vaccine vs placebo), adjusted for herpes simplex virus 2 status at Phambili enrollment, within subgroups.

	Phambili follow-up		Phambili + 503-S follow-up	
	HR (95% CI)	p-value	HR (95% CI)	p-value
**Overall**	1.70 (1.14, 2.56)	0.01	1.52 (1.08, 2.15)	0.02
**Sex**		0.19 ^a^		0.02 ^a^
Women	1.42 (0.85, 2.35)	0.18	1.12 (0.73, 1.72)	0.62
Men	2.46 (1.22, 4.93)	0.01	2.75 (1.49, 5.06)	0.001
**Circumcision status**		0.48 ^a^		0.43^a^
Men circumcised at baseline or on study	3.25 (1.06, 9.98)	0.04	3.69 (1.38, 9.89)	0.01
Uncircumcised men	1.99 (0.81, 4.90)	.14	2.27 (1.03, 5.02)	0.04
**Ad5 serostatus**		0.98 ^a^		0.75 ^a^
Ad5 seropositive	1.70 (1.08, 2.67)	0.02	1.57 (1.07, 2.31)	0.02
Ad5 seronegative	1.70 (0.66, 4.40)	0.27	1.37 (0.64, 2.94)	0.41
**Age**		0.95 ^a^		0.96 ^a^
Age ≤ 25 years	1.72 (1.04, 2.84)	0.03	1.51 (0.99, 2.30)	0.055
Age > 25 years	1.66 (0.83, 3.32)	0.15	1.54 (0.85, 2.80)	0.15
**Study site**		0.50 ^a^		0.50 ^a^
Soweto	2.39 (1.18, 4.83)	0.02	2.00 (1.10, 3.64)	0.02
Cape Town	1.86 (0.84, 4.14)	0.13	1.78 (0.86, 3.68)	0.12
KOSH	0.87 (0.33, 2.25)	0.77	1.03 (0.51, 2.08)	0.94
eThekwini	1.91 (0.62, 5.94)	0.26	1.83 (0.68, 4.91)	0.23
Medunsa	1.04 (0.21, 5.21)	0.96	0.73 (0.16, 3.27)	0.68

HR = hazard ratio (vaccine vs placebo), adjusted for baseline herpes simplex virus 2 status. Ad5 = adenovirus serotype 5.

^a^ p-value for test of interaction for subgroup and treatment group

**Fig 2:**
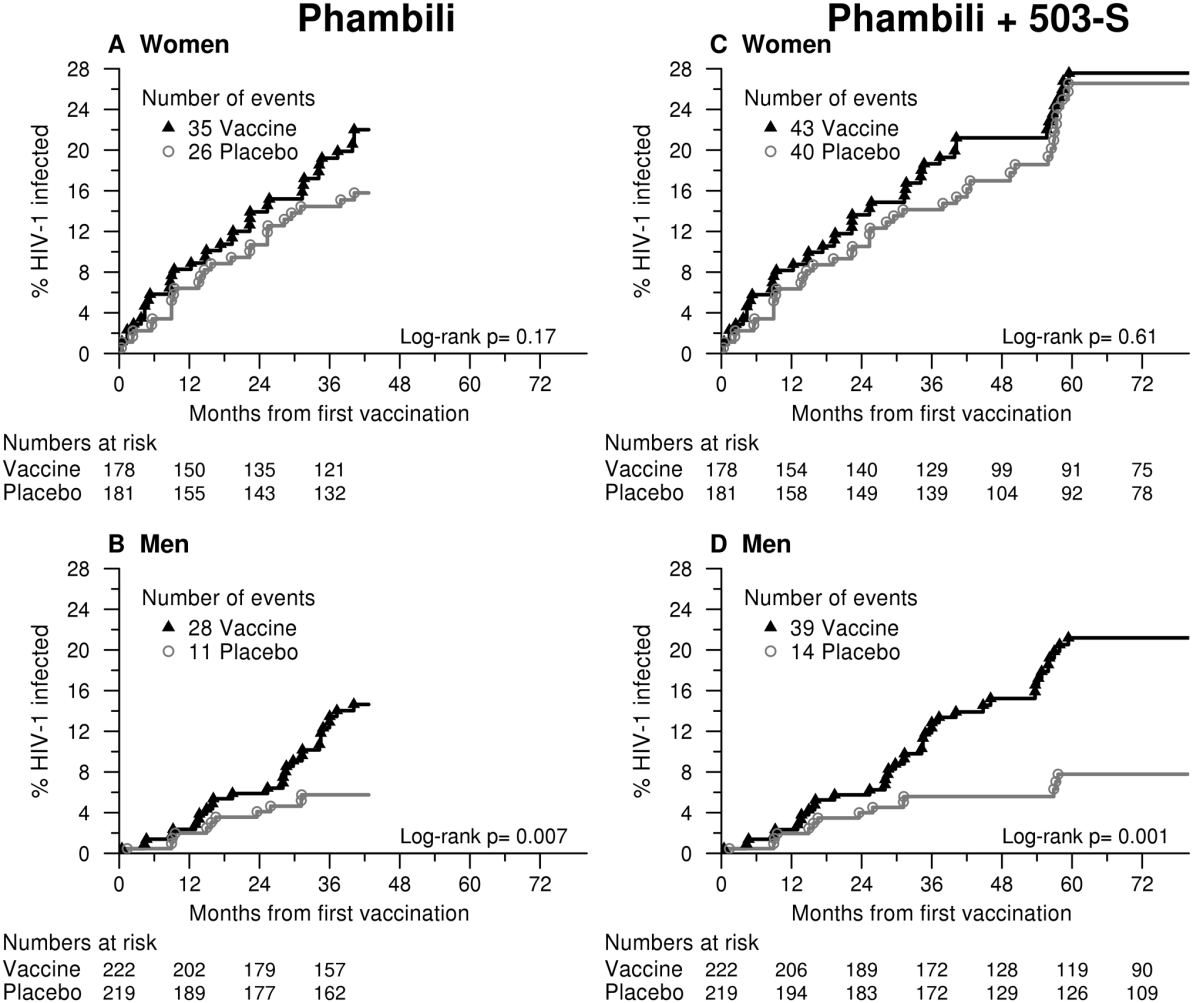
Cumulative HIV-1 incidence curves for vaccine and placebo groups for Phambili and Phambili + HVTN 503-S follow-up by gender.

With the additional HVTN 503-S follow-up, the vaccine:placebo HRs were significantly different for men and women (interaction p = 0.02, table 3). The effect on HIV-1 acquisition by vaccination status was more pronounced in men [HR = 2.75, 95% CI: (1.49, 5.06)], and less for women [HR = 1.12, 95% CI: (0.73, 1.72)], Fig 2. There was no evidence that the HR varied by baseline Ad5 status, age, study site or, in men, by time-dependent circumcision status (interaction p-values ≥ 0.42, table 3). Sites promoted male circumcision as an HIV prevention measure. Although men may have been at increased risk of HIV infection in the weeks following circumcision, there were few HIV-1 infections diagnosed within 3 months following circumcision. The 14 men circumcised at enrollment who later acquired HIV had been circumcised a minimum of 8 months prior to enrollment into Phambili. Among the 11 men who were circumcised after enrollment and became HIV-1 infected, 7 had an HIV negative test at least 2 months after their circumcision and the other 4 were HIV seropositive at their first post-circumcision HIV test between 3–6 months post-circumcision (they had no HIV seronegative test after circumcision).

Of the 441 men enrolled in Phambili, 271 (61%) were categorized as completely circumcised either at enrollment or during follow-up (up to and including final Phambili study visit), (table 4). For half of these men (134/271), circumcision status was based on visual exam; for the other half, self-report was accepted (136/271); and 1 circumcised man was missing data on Phambili assessment method. The remaining 170 men were categorized as uncircumcised, primarily based on self-report (159, 94%); only 11 (6%) underwent visual exam of their circumcision status at their final Phambili assessment.

**Table 4:** Comparison for men of circumcision status from Phambili to HVTN 503-S assessment (N = 441).

Phambili Assessment	Phambili Assessment Method	Total for Phambili	503-S Circumcision Assessment (physical examination)				No 503-S Circumcision Assessment	
			Circumcised at Phambili enrollment	Circumcised during Phambili follow-up	Circumcised between end of Phambili follow-up and 503-S visit	Uncircumcised		
Circumcised at Phambili enrollment		129	62	0	0	4^a^	1	62
	Physical examination	79	39	0	0	2 ^b^	0	38
	Self-report	50	23	0	0	2 ^b^	1	24
Circumcised during Phambili follow-up		142	0	93^c^	0	3^d^	12	34
	Physical examination	55	0	41	0	3 ^b^	1	10
	Self-report	86	0	51	0	0 ^b^	11	24
Uncircumcised throughout Phambili follow-up		162	2	6	10	60	6	78
	Physical examination	9	0	0	1	2	0	6
	Self-report	153	2 ^b^	6 ^b^	9	58	6	72
Uncircumcised at Phambili enrollment but no follow-up circumcision assessment		8	0	0	0	0	0	8
	Physical examination	2	0	0	0	0	0	2
	Self-report	6	0	0	0	0	0	6
**Total**		441	64	99	10	67	19	182

^a^ All had partial circumcisions.

^b^ Participants with discordant circumcision status data between Phambili and 503-S (i.e., Phambili assessment was incorrect): 6% (15/240)

^c^1 man circumcised during Phambili follow-up was missing data on Phambili assessment method.

^d^ 2 of the 3 had partial circumcisions.

259 of the 441 Phambili men (59%) enrolled in HVTN 503-S and 240 (92%) of these agreed to undergo physical examination to verify their circumcision status (S2 Fig). Of those men whose circumcision status was verified by physical examination in HVTN 503-S, 94% (225/240) were found to have been correctly categorized in Phambili. The remaining 15 men (6%) who underwent physical examination in HVTN 503-S were found to have had their circumcision status mis-categorized in Phambili.

The majority (83%) of enrolled participants correctly recalled their treatment assignment, with no difference in recall between treatment groups, HIV-infection status, or by gender. Those who had discontinued Phambili follow-up early (n = 70) were less likely to correctly remember their treatment assignment than those who completed follow-up (n = 394): 18.6% vs 8.4%.

Most participants reported no change in frequency of sex (75.4%), number of sexual partners (79.7%) or condom use (78.8%) between the year before and year after their last Phambili visit (table 5). There were no differences in these self-reported risk behaviors by Phambili treatment group (table 5) or follow-up status (data not shown). Women were more likely than men to report no change (table 5). Borderline differences between treatment groups were seen for men with number of partners (p = 0.054), with placebo recipients more likely to report an increase in number of partners (16.4%) than vaccinees (6.9%) and for women, placebo recipients more likely to report a decrease in condom use (17.0%) than vaccinees (6.1%) (p = 0.052).

**Table 5:** Change in self-reported risk behavior between one year before and one year after last Phambili visit.

	All (N = 463)		Vaccine (N = 229)		Placebo (N = 234)			Men (N = 258)		Women (N = 205)		
	N	%	N	%	N	%	p-value	N	%	N	%	p-value
**Frequency of sex**							0.75					0.047
Decreased	50	10.8	27	11.8	23	9.8		35	13.6	15	7.3	
No change	349	75.4	172	75.1	177	75.6		184	71.3	165	80.5	
Increased	64	13.8	30	13.1	34	14.5		39	15.1	25	12.2	
**Number of partners**							0.11					< 0.001
Decreased	55	11.9	27	11.8	28	12.0		42	16.3	13	6.4	
No change	368	79.7	188	82.5	180	76.9		186	72.1	182	89.2	
Increased	39	8.4	12	5.7	26	11.1		30	11.6	9	4.4	
**Condom use**							0.37					0.005
Decreased	52	11.2	21	9.2	31	13.2		28	10.9	24	11.7	
No change	365	78.8	184	80.3	181	77.4		194	75.2	171	83.4	
Increased	46	9.9	24	10.5	22	9.4		36	14	10	4.9	

One man who received placebo and one woman who received vaccine are missing risk behavior data and an additional female vaccinee is missing change in number of partners.

## Discussion

The additional data from the extended follow-up of participants in HVTN 503-S substantiated the finding of increased HIV-1 acquisition among vaccinated Phambili men. The recall study also strengthened the evidence of a lack of vaccine effect among women, where the high HIV-1 incidence seen in women implied sufficient power for this evaluation. Due to the uncertainty in the timing of the infections diagnosed in HVTN 503-S (approximately 3 years between the last seronegative test in Phambili and the seropositive test in HVTN 503-S), we cannot determine whether the increased risk of acquisition seen in men is sustained at the time of the HVTN 503-S or whether most of the infections among vaccinated men occurred shortly after their last Phambili visit. A biological mechanism to explain the increased risk associated with the recombinant adenovirus 5 vectored polyvalent HIV-1 *gag/pol/nef* subtype B vaccine has not been identified. Vaccination may have led to an increased number of activated Ad5-specific T cells that then served as target cells for HIV-1 [18–21].

Another possible explanation is that vaccine-induced subclinical inflammation may have contributed to the increased rate of HIV-1 infection in men. Vaccine-induced subclinical inflammation may not have contributed to any additional risk of HIV-1 acquisition for women in the trial given the high levels of genital inflammation already present in the general population of South African women [22].

The HVTN 503-S finding of increased risk of HIV-1 acquisition among vaccinated men supports the Step results. However, unlike Step, we did not see differences in HIV-1 risk due to the vaccine among subgroups of circumcised and uncircumcised men or in those with vs. without prior immunity to Ad5. This may be due to differences in study population: the Step cohort consisted mainly of MSM in the Americas, Caribbean, and Australia whereas the Phambili cohort consisted mainly of South African heterosexuals having vaginal sex and a higher proportion of participants who were Ad5 seropositive at enrollment (81%).

While enrollment into HVTN 503-S was high, 33% of the targeted Phambili participants did not enroll. This was mostly due to inability to contact participants, unsurprising given the young study population and the long interval (almost 3 years) between last contact in Phambili and the start of the recall study and the high rates of migration in some of the trial communities. Enrollment rates differed by site but not by the following significant predictors of HIV-1 acquisition in Phambili: treatment group, gender, or baseline HSV-2 status. Therefore the participants enrolled in the recall study were well matched to those who did not enroll on the key known risk factors in the Phambili study population. Nonetheless, the recall study was not able to enroll a third of the eligible uninfected Phambili participants and it is possible that these participants differed with respect to unmeasured risk factors from those who did enroll.

In addition to supporting the overall Phambili findings, the recall study has identified challenges in collecting circumcision data in a population where self-reported data may be unreliable and on-study circumcision rates are high. Our concerns regarding accuracy of the circumcision status assessment in Phambili were warranted. In HVTN 503-S, 6% of men who accepted a physical examination had discrepant circumcision status between Phambili and HVTN 503-S due to inaccurate self-report and incorrect physical examination assessments. This indicates that better training is needed in both of these areas including guidance on distinguishing partial circumcisions and the importance of verifying self-reporting uncircumcised men by physical examination. Partial circumcisions were classified as uncircumcised in the risk of acquisition analyses given data indicating that the presence of the inner foreskin fold may increase the risk of HIV entry [23]. Among the 19 men who declined physical examination, 7 were from one site and represented half of the men enrolled by the site. During the conduct of HVTN 503-S, the site identified that this was due in part to a staff member who was uncomfortable performing a genital exam. For future trials in this region in which male circumcision status has the potential to affect the evaluation of the efficacy of the study intervention, we recommend that physical examinations be done by staff who are trained in determining circumcision status and in communicating to participants the importance of a visual exam. Consideration should also be given to repeat assessment of circumcision status if it is expected to change throughout the trial for a substantial proportion of men.

The high proportion of correct recall of Phambili treatment assignment among HVTN 503-S participants, with no differences by treatment group or by gender, indicates that sites were informing participants clearly of their treatment assignment and that the information was well retained.

Finally, the HVTN 503-S recall study demonstrated the skill and dedication of site staff in locating and enrolling a large proportion of Phambili participants almost 3 years after the end of the Phambili study. Staff succeeded in enrolling 38% of participants who had dropped out of Phambili several years before; this speaks to the commitment of the participants, community advisory board members, and the site staff. Through their efforts, HVTN 503-S provided additional data that supported the results of the analysis of the Phambili primary objectives.

## Supporting Information

S1 Fig Consort diagram of participant flow. (TIFF)

S2 Fig Consort diagram of evaluation of circumcision status among men. (TIFF)

S1 Protocol (PDF)

S1 TREND Checklist TREND checklist. (PDF)
