# Supplementary figures and images for: Continued Follow-Up of Phambili Phase 2b Randomized HIV-1 Vaccine Trial Participants Supports Increased HIV-1 Acquisition among Vaccinated Men
Source: PLoS One. 2015 Sep 14;10(9):e0137666. doi: 10.1371/journal.pone.0137666 (PMC4569275; doi:10.1371/journal.pone.0137666)

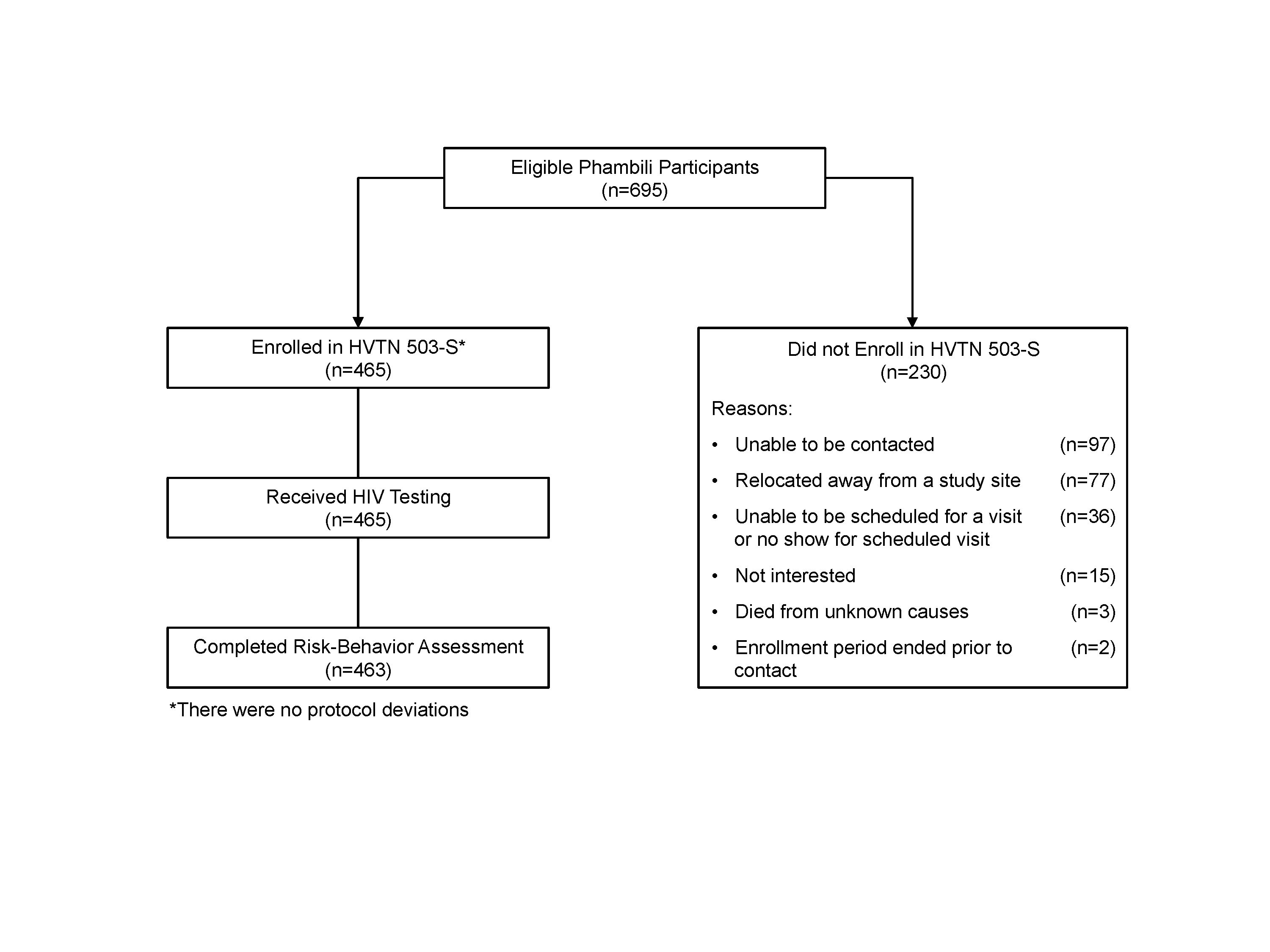

Supplement: S1 Fig — (TIFF) [file pone.0137666.s001.tiff]

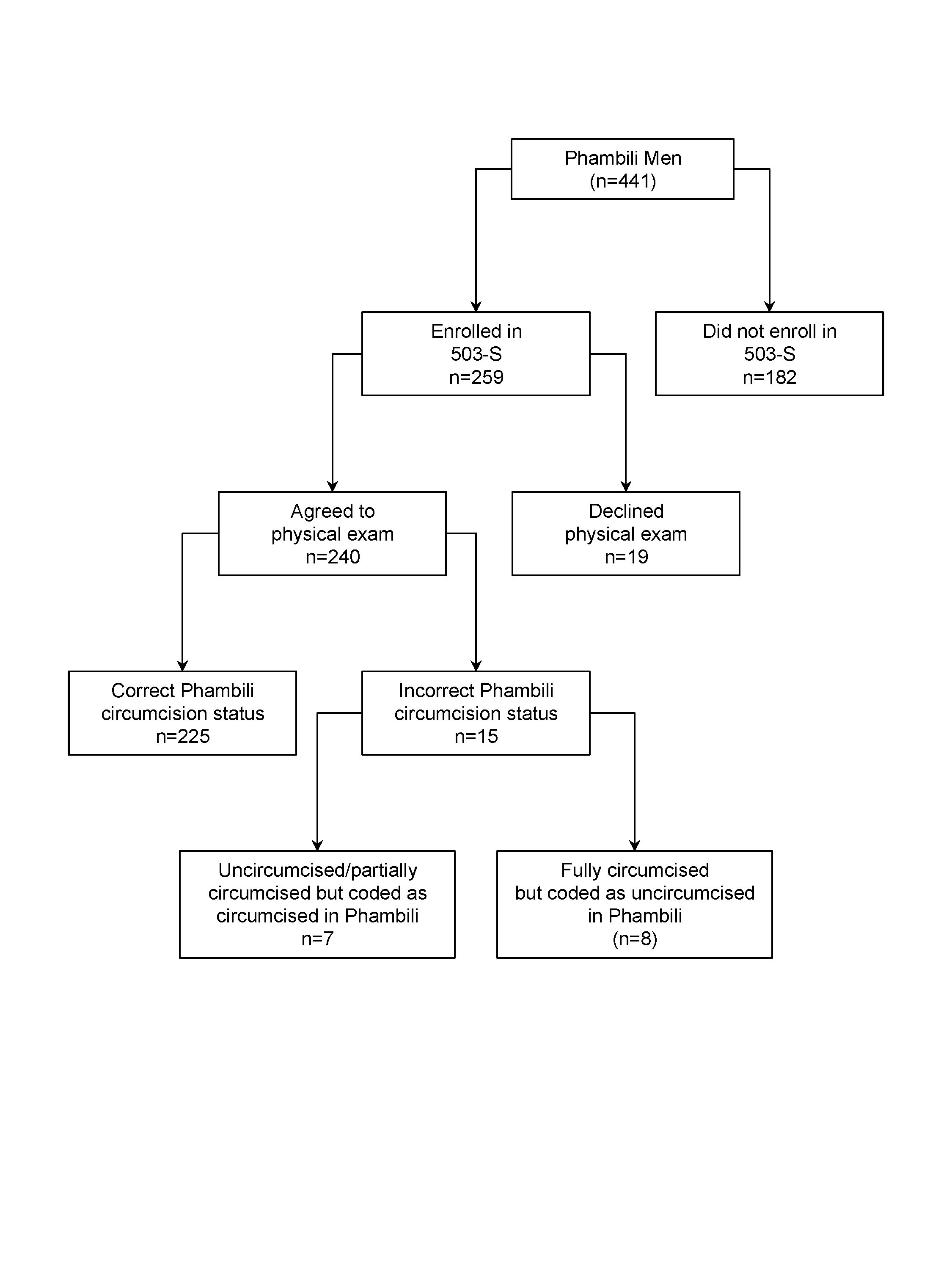

Supplement: S2 Fig — (TIFF) [file pone.0137666.s002.tiff]
