# Supplementary material for: Continued Follow-Up of Phambili Phase 2b Randomized HIV-1 Vaccine Trial Participants Supports Increased HIV-1 Acquisition among Vaccinated Men
Source: PLoS One. 2015 Sep 14;10(9):e0137666. doi: 10.1371/journal.pone.0137666 (PMC4569275; doi:10.1371/journal.pone.0137666)
Supplement: S1 Protocol — (PDF) [file pone.0137666.s003.pdf]

HIV VACCINE
TRIALS NETWORK

Sub-study

HVTN 503-S

HIV testing and behavioral risk assessment for former HVTN 503 (Phambili) participants

DAIDS DOCUMENT ID 11949

Parent Protocol

HVTN 503 (DAIDS ID 10392): A multicenter double-blind randomized placebo-controlled Phase IIB test-of-concept study to evaluate the safety and efficacy of a 3-dose regimen of the Clade B-based Merck Adenovirus serotype 5 HIV-1 gag/pol/nef vaccine in HIV-1-uninfected adults in South Africa

BB IND 13028 HELD BY DAIDS

CLINICAL TRIAL SPONSORED BY

Division of AIDS (DAIDS)
National Institute of Allergy and Infectious Diseases (NIAID)
National Institutes of Health (NIH)
Department of Health and Human Services (DHHS)
Bethesda, Maryland, USA

April 25, 2013
FINAL
HVTN 503-S, Version 1.0

Contents

1 Ethical considerations 3

2 IRB/EC review considerations 5
- 2.1 Minimized risks to participants 5
- 2.2 Reasonable risk/benefit balance 5
- 2.3 Equitable subject selection 6
- 2.4 Appropriate informed consent 6
- 2.5 Protect privacy/confidentiality 6

3 Overview 7
- 3.1 Protocol Team 8

4 Background and rationale 9

5 Objectives and endpoints 11

6 Statistical considerations 12

7 Clinical procedures 13
- 7.1 Informed consent 13
- 7.2 Physical examination 13
- 7.3 Confirm unblinding information 13
- 7.4 HIV counseling and testing 13
- 7.5 Behavioral risk assessment 14
- 7.6 Specimen collection 14
- 7.7 Follow-up visit 14
- 7.8 Post-study contact 14
- 7.9 Visit windows 15
- 7.10 VISP registry consent form 15

8 Laboratory 16
- 8.1 Exploratory studies 16
- 8.2 Other use of stored specimens 16
- 8.3 Biohazard containment 16

9 Protocol conduct 18

10 Document references (other than literature citations) 19

11 Acronyms and abbreviations 20

12 Literature cited 21

Appendix A Sample informed consent form 22

Appendix B Sample consent form for use of samples and information in other studies 28

Appendix C HVTN VISP registry consent 32

Appendix D Laboratory procedures (placebo recipients) 35

Appendix E Laboratory procedures (vaccinees) 36

Appendix F Procedures at HVTN CRS 37

1 Ethical considerations

The HIV Vaccine Trials Network (HVTN) has addressed ethical concerns in the following ways:

- HVTN scientists and operational staff incorporate the philosophies underlying major codes [1-3], declarations, and other guidance documents relevant to human subjects research into the design and conduct of HIV vaccine clinical trials.

- HVTN scientists and operational staff are committed to substantive community input—into the planning, conduct, and follow-up of its research—to help ensure that locally appropriate cultural and linguistic needs of study populations are met. Community Advisory Boards (CAB) are required by DAIDS and supported at all HVTN research sites to ensure community input.

- HVTN clinical trial staff counsel study participants routinely on how to reduce HIV risk. Participants who become HIV-infected during the trial are provided counseling on notifying their partners and about HIV infection according to local guidelines. Staff members will also counsel them about reducing their risk of transmitting HIV to others.

- The HVTN requires that all international HVTN sites lacking national plans for providing antiretroviral therapy (ART) develop plans for the care and treatment of participants who acquire HIV infection during a trial. Each plan is developed in consultation with representatives of host countries, communities from which potential trial participants will be drawn, sponsors, and the HVTN. Participants will be referred to programs for ART provision when the appropriate criteria for starting ART are met. If a program is not available at a site and ART is needed, a privately established fund will be used to pay for access to treatment to the fullest extent possible.

- The HVTN provides training so that all participating sites similarly ensure fair participant selection, protect the privacy of research participants, and obtain meaningful informed consent. During the study, participants will have their wellbeing monitored, and to the fullest extent possible, their privacy protected. Participants may withdraw from the study at any time.

- Prior to implementation, HVTN trials are reviewed by scientists who are not involved in the conduct of the trials under consideration.

- HVTN trials are reviewed by local and national regulatory bodies and are conducted in compliance with all applicable national and local regulations.

- The HVTN designs its research to minimize risk and maximize benefit to both study participants and their local communities. For example, HVTN protocols provide enhancement of participants' knowledge of HIV and HIV prevention, as well as counseling, guidance, and assistance with any social impacts that may result from research participation. HVTN protocols also include careful medical review of each research participant's health conditions and reactions to study products while in the study.

- HVTN research aims to benefit local communities by directly addressing the health and HIV prevention needs of those communities and by strengthening the capacity of the communities through training, support, shared knowledge, and equipment. Researchers involved in HVTN trials are able to conduct other critical research in their local research settings.

- The HVTN recognizes the importance of institutional review and values the role of in country Institutional Review Boards (IRBs) and Ethics Committees (ECs) as custodians responsible for ensuring the ethical conduct of research in each setting.

2 IRB/EC review considerations

United States (US) Food and Drug Administration (FDA) and other US federal regulations require IRBs/ECs to ensure that certain requirements are satisfied on initial and continuing review of research (Title 45, Code of Federal Regulations (CFR), Part 46.111(a) 1-7; 21 CFR 56.111(a) 1-7). The following section highlights how this protocol addresses each of these research requirements. Each HVTN Investigator welcomes IRB/EC questions or concerns regarding these research requirements.

This study is being conducted exclusively in South Africa, with funding from the U. S. NIH. Due to this, the trial is subject to both US and South African regulations and guidelines on the protection of human research subjects and ethical research conduct. These research regulations and guidelines are based on ethical principles of respect for persons, beneficence and nonmaleficence, and justice. Where there is a conflict in regulations or guidelines, the regulation or guideline providing the maximum protection of human research subjects will be followed.

In compliance with the Guidelines For Good Practice In The Conduct Of Clinical Trials In Human Participants In South Africa (“South African GCPs”), each research location in South Africa has a South African-based Principal Investigator (PI) who is qualified to conduct (and supervise the conduct of) the research; and the research addresses an important South African health need for an HIV vaccine in line with the national strategic plan for South Africa and the national South African HIV vaccine plan. The investigators take responsibility for the conduct of the study, including obtaining all appropriate South African regulatory and ethical reviews of the research. Each participating site has a standard operating procedure for ensuring that participants have the necessary information to make a decision whether or not to consent to the research. The sections below address each of the review concerns by IRBs/ethics committees ECs regarding how the research will be conducted.

2.1 Minimized risks to participants

45 CFR 46.111 (a) 1 and 21 CFR 56.111 (a) 1: Risks to subjects are minimized.

This protocol minimizes risks to participants by (a) correctly and promptly informing participants about risks so that they can join in partnership with the researcher in recognizing and reporting harms; (b) respecting local/national blood draw limits; (c) having staff properly trained in administering study procedures that may cause physical harm or psychological distress, such as blood draws, HIV testing and counseling and HIV risk reduction counseling; and (e) providing HIV risk reduction counseling.

2.2 Reasonable risk/benefit balance

45 CFR 46.111 (a) 2 and 21 CFR 56 (a) 2: Risks to subjects are reasonable in relation to anticipated benefits, if any, to subjects, and the importance of the knowledge that may reasonably be expected to result.

In all public health research, the risk-benefit ratio may be difficult to assess because the benefits to a healthy participant are not as apparent as they would be in treatment

protocols, where a study participant may be ill and may have exhausted all conventional treatment options. However, this protocol is designed to minimize the risks to participants while maximizing the potential value of the knowledge it is designed to generate.

2.3 Equitable subject selection

45 CFR 46.111 (a) 3 and 21 CFR 56.111 (a) 3: Subject selection is equitable

This protocol has specific inclusion and exclusion criteria for investigators to follow in admitting participants into the protocol. Participants are selected because of these criteria and not because of positions of vulnerability or privilege. Investigators are required to maintain screening and enrollment logs to document volunteers who screened into and out of the protocol and for what reasons.

2.4 Appropriate informed consent

45 CFR 46.111 (a) 4 & 5 and 21 CFR 56.111 (a) 4 & 5: Informed consent is sought from each prospective subject or the subject's legally authorized representative as required by 45 CFR 46.116 and 21 CFR Part 50; informed consent is appropriately documented as required by 45 CFR 46.117 and 21 CFR 50.27

The protocol specifies that informed consent must be obtained before any study procedures are initiated and assessed throughout the trial (see Section 7.1). Each site is provided training in informed consent by the HVTN as part of its entering the HVTN. The HVTN requires a signed consent document for documentation, in addition to chart notes or a consent checklist.

2.5 Protect privacy/confidentiality

45 CFR 46.111 (a) 7 and 21 CFR 56.111 (a) 7: There are adequate provisions to protect the privacy of subjects and maintain the confidentiality of data.

Privacy refers to an individual's right to be free from unauthorized or unreasonable intrusion into his/her private life and the right to control access to individually identifiable information about him/her. The term "privacy" concerns research participants or potential research participants as individuals whereas the term "confidentiality" is used to refer to the treatment of information about those individuals. This protocol respects the privacy of participants by informing them about who will have access to their personal information and study data (see Appendix A). The privacy of participants is protected by assigning unique identifiers in place of the participant's name on study data and specimens. In addition, each staff member at each study site in this protocol signs a Confidentiality Agreement with the HVTN and each study site participating in the protocol is required to have a standard operating procedure on how the staff members will protect the confidentiality of study participants.

3 Overview

Title

HIV testing and behavioral risk assessment for former HVTN 503 (Phambili) participants

Primary objective(s)

To expand HIV diagnostic testing coverage among the HVTN 503 (Phambili) study cohort

To expand information on behavioral risk among the HVTN 503 (Phambili) study cohort

Participants

Phambili study participants whose HIV status was “uninfected” at the time of termination from Phambili (~700) will be invited to enroll.

Duration per participant

2 visits to occur approximately 2 weeks apart plus an additional contact to provide study results to participants

Estimated total study duration

Enrollment and study-visits to a maximum of 6 months following protocol activation for each Phambili study site. Total study duration (from first site activation to 6 months after final site activation) estimated at 8 months.

Investigational New Drug (IND) sponsor

DAIDS, NIAID, NIH, DHHS (Bethesda, Maryland, USA)

Core operations

HVTN Vaccine Leadership Group/Core Operations Center, Fred Hutchinson Cancer Research Center (FHCRC) (Seattle, Washington, USA)

Statistical and data management center (SDMC)

Statistical Center for HIV/AIDS Research and Prevention (SCHARP), FHCRC (Seattle, Washington, USA)

HIV diagnostic laboratory

Serology-Virology & Special Molecular Diagnostic Unit, National Institute for Communicable Diseases (Johannesburg, South Africa)

Study sites

HVTN 503 (Phambili) study sites

3.1 Protocol Team

Protocol leadership

Chair	Glenda Gray<br>Chris Hani Baragwanath Hospital<br>27-11-989-9702/615-8466<br>gray@pixie.co.za	*Statistician*	Zoe Moodie<br>SCHARP, FHCRC<br>206-667-7077<br>zoe@scharp.org
Cochair	Jim Kublin<br>FHCRC<br>206-667-1970<br>jkublin@fhcrc.org	*Medical officer*	Mary Allen<br>DAIDS, NIAID<br>301-402-2310<br>mallen@niaid.nih.gov

Other contributors

Vaccine developer representative	Michael Robertson<br>Merck and Co., Inc.	*Clinical trials manager*	Niles Eaton<br>HVTN Core, FHCRC
Laboratory Program representative	Eva Chung<br>HVTN Laboratory Program, FHCRC	*HVTN program manager*	Gina Escamilla<br>SCHARP, FHCRC
Regulatory affairs	Renée Holt<br>HVTN Core, FHCRC	*Protocol development coordinator*	Carter Bentley<br>HVTN Core, FHCRC
Statistical research associate	Barb Metch<br>SCHARP, FHCRC	*HVTN social scientist*	Michele Peake Andrasik<br>HVTN Core, FHCRC
Community engagement unit rep	Genevieve Meyer<br>HVTN Core, FHCRC		

4 Background and rationale

Phambili (HVTN 503) was a phase 2b proof-of-concept study of the Merck MRKAd5 HIV-1 *gag/pol/nef* vaccine among healthy, sexually active, 18-35 year old volunteers in the Republic of South Africa. In addition to testing vaccine safety and tolerability, the Phambili trial was designed to evaluate vaccine efficacy in preventing HIV-1 infection and also in reducing the setpoint viral load of study participants who became HIV-1–infected. Phambili was initiated to evaluate potential cross-clade activity of the clade B Merck vaccine in a clade C region, while the vaccine was being evaluated concurrently in the Step study (Merck 023/HVTN 502) in regions where the circulating HIV-1 strains were predominantly clade B.

The Step study began enrolling in December 2004 and Phambili began enrolling on 24 January 2007. In September 2007, following a Data and Safety Monitoring Board finding that the Step study had met preset criteria for futility in terms of its primary efficacy objective, vaccinations in that study were stopped and study participants were unblinded, though participant follow-up continued and was extended further through HVTN 504 [4,5]. Subsequent analysis of the Step cohort showed that among subgroups of male participants defined by pre-existing immunity to the adenovirus type 5 (Ad5) vaccine vector and/or who were uncircumcised, vaccine recipients had a statistically significant increased risk of HIV-1 acquisition. The Step male cohort comprised primarily men who had sex with men. At the time enrollment in Step was stopped, there were too few HIV infections among female participants to evaluate vaccine effects on risk of HIV infection in this subgroup. Further follow-up of the Step cohort over more than 4 years, with retention of more than 80% of eligible participants, demonstrated that the increased acquisition effect in men waned with time from vaccination [5].

When futility in the Step study was reached, enrollment was suspended in the Phambili study, vaccinations were discontinued, and the 801 enrolled study participants were unblinded. The protocol was amended to continue following HIV-uninfected study participants for 3.5 years after their initial vaccination, with clinic visits and HIV testing every 3 months. An interim analysis of this study, conducted after an average of 22.5 months of participant follow-up, did not find any increased risk of HIV acquisition in the vaccinated group [6].

Initial analysis following completion of follow-up in the Phambili study has recently been completed. Of the 400 study participants assigned to receive vaccine, 112 received 1 vaccination, 259 received 2 vaccinations, and only 29 received the planned regimen of 3 vaccinations. At the time enrollment and vaccinations were stopped, study participants had received an average of 4.3 months of follow up. Analysis of blinded follow-up showed no vaccine effect on HIV acquisition at that point. Over the entire follow-up period, among the 800 participants not infected at enrollment, 100 became HIV-infected, 63 in the vaccine arm and 37 in the placebo arm: a 1.7 fold increased relative risk for HIV acquisition, adjusted for baseline HSV-2 status. Increased acquisition of HIV in the vaccine group appears more pronounced in men than women, and is more evident after 30 months of participant follow up than in the first 18 months of follow up. At approximately 30 months after enrollment there appeared to be a widening difference between the vaccine and placebo groups due to a flattening of the HIV acquisition rate in placebo recipients (especially male placebo recipients) and continued HIV acquisition in vaccinated subjects. In this analysis, neither circumcision status nor prior antibodies to Ad5 modified the vaccine effect on HIV infection.

Although the vaccine effect on HIV acquisition over the entire HVTN 503 study follow-up period is statistically significant, the vast majority of follow-up occurred after unblinding, introducing the potential for bias that could substantially influence these data. For instance, if higher risk placebo recipients were more likely to drop out or vaccinated participants engaged in higher risk behavior, then the difference in risk between vaccine and placebo groups could be significantly exaggerated.

In order to better understand these data, participants who were not diagnosed with HIV infection during their participation in Phambili will be recalled to their study sites to receive information about the study and to be offered HIV counseling and testing. The participants will also be offered enrollment in this sub=study in order to record HIV test results as data, to collect blood samples for possible laboratory testing in the future, and to collect additional data on behavioral risk.

Self-report data on sexual behavior will be collected to address the following 2 questions:

1. Does recalled HIV risk behavior differ in vaccine and placebo groups, particularly in last year of Phambili?

2. Do participants who dropped out recall having more frequent HIV risk behavior than those that did not drop out, particularly in last year of Phambili?

The extended recall period over which self-reported sexual behavior will be collected (2-4 years in many cases) makes the reliability of these data potentially problematic. To address these difficulties, participants will be asked about behavior during their weeks of maximum sexual activity (hence potential greatest risk of HIV acquisition) during the last year of their participation in Phambili and in the year after their last Phambili study visit. Participants will be asked their frequency of unprotected sex during weeks of maximum sexual activity. They will be asked whether they think any of their partners during those periods were HIV-infected. In addition, changes in sexual behavior after participation in Phambili ended will be elicited, along with STI diagnoses post-Phambili. In order to detect the possibility of sexual networks and potential infection clusters associated with Phambili participation, study participants will be asked whether they ever had sex with someone they met at the Phambili clinic. Memory aids of known effectiveness, such as calendars, significant events (election, major news stories, new job, relationship, anniversary, birthday, deaths, etc.), and anchor dates will be employed to maximize recall acuity of those specific periods.

5 Objectives and endpoints

Primary objective 1:

To expand HIV diagnostic testing coverage among the HVTN 503 (Phambili) study cohort

Primary endpoint 1:

HIV-1 infection

Primary objective 2:

To gather additional information on behavioral risk among the HVTN 503 (Phambili) study cohort

Primary endpoint 2:

Self-report of sexual behaviors associated with risk of HIV acquisition

6 Statistical considerations

The primary objective of expanding HIV diagnostic testing coverage among the HVTN 503 study cohort may provide additional HIV infection endpoints to contribute to the analysis of the vaccine effect on HIV acquisition. Note that, in addition to laboratory-based diagnoses (and confirmations) of HIV infection during this sub-study, self-reports of HIV infection by placebo recipients will be accepted as endpoint data. The primary analysis of this sub-study will use the modified intent-to-treat (MITT) study population, which includes all vaccinated participants apart from those determined to be HIV-1 infected on the first day of vaccination. Cox proportional hazards models will be used to estimate the infection hazard ratio (HR) for vaccine to placebo, adjusting for covariates as appropriate, with Wald-based 95% confidence intervals. Interval-censored methods will be considered to account for the variability across participants' last HIV-uninfected and first HIV-infected test dates.

The second primary objective involving the collection of additional information on behavioral risk among Phambili participants may improve estimates of HIV exposure in vaccine and placebo groups to aid in understanding whether there was differential exposure among treatment groups possibly due to unblinding or differential dropout.

The major focus of the behavioral analysis will be to assess differences in the self-reported, recalled number of unprotected sex partners and frequency of unprotected sex during the week of most sexual activity in the last year of participation in Phambili. Depending on the distributions of these data, categorical, Wilcoxon rank sum or two sample t-test statistical tests will be used to assess differences between groups. Comparisons will be made between the vaccine and placebo groups and between those who completed and did not complete their Phambili study visits.

7 Clinical procedures

The schedule of clinical procedures is shown in Appendix F .

7.1 Informed consent

The HVTN informed consent forms (see Appendix A and Appendix B) document that a participant (1) has been informed about the potential risks, benefits, and alternatives to participation, and (2) is willing to participate in an HVTN study. HVTN clinical research site (CRS) staff will obtain informed consent of participants according to HVTN policies and procedures.

Informed consent must be obtained prior to performing any of the study procedures listed below.

Regarding protocol registration, sites should follow procedures outlined in the current version of the DAIDS Protocol Registration Manual.

7.2 Physical examination

At the enrollment visit, a CRS clinician will perform a complete physical examination, including height, weight, vital signs; clinical assessments of head, ears, eyes, nose, and throat; neck; lymph nodes; heart; chest; abdomen; extremities; neurological function; and skin. For male participants, this examination will include an assessment of circumcision status, documentation of whether circumcision is partial or complete, traditional or medical male circumcision, and approximate date of circumcision (see HVTN 503-S Study Specific Procedures).

7.3 Confirm unblinding information

The clinic staff will elicit the participant's recall of the treatment assignment (vaccine or placebo) given at unblinding, and then will confirm that the participant has the correct treatment assignment information.

7.4 HIV counseling and testing

At the first clinic visit, HIV counseling will be performed in compliance with the US Centers for Disease Control and Prevention (CDC) guidelines or other local guidelines for HIV counseling and referral.

For participants who have been diagnosed with HIV infection at a Phambili site prior to enrolling in this sub-study, additional HIV testing is not required and documentation of diagnosis will be obtained from that site. If a participant who received placebo was diagnosed with HIV infection at a site not associated with Phambili, they will be asked for their approximate date of diagnosis of HIV infection and additional HIV testing is not required. Participants who received vaccine but were diagnosed at a site not associated

with Phambili are at risk of being falsely diagnosed as HIV infected due to vaccine-induced seropositivity (VISP). For participants who received vaccine and were diagnosed as HIV infected at a site not associated with Phambili, CRS staff should follow procedures outlined in Appendix E to confirm HIV infection status.

For participants who have not been diagnosed as HIV-infected, HIV testing will be performed as follows:

- For placebo recipients, HIV testing will be performed as indicated in Appendix D.[a]

- For vaccine recipients, HIV testing will be performed in accordance with the current HVTN HIV testing algorithm (see HVTN Site Lab Reference Manual), which is able to distinguish vaccine-induced antibody responses from actual HIV infections (see Appendix E).

During the physical examination, signs or symptoms of an acute HIV infection syndrome, an intercurrent illness consistent with HIV-1 infection, or probable HIV exposure would prompt a diagnostic workup per the HVTN algorithm for Recent Exposure/Acute Infection Testing to determine HIV infection.

Participants will also be counseled on HIV prevention.

7.5 Behavioral risk assessment

A questionnaire concerning the occurrence and frequency of behaviors associated with risk of HIV acquisition will be administered.

7.6 Specimen collection

Blood samples for HIV diagnostic testing and for exploratory studies yet to be determined will be collected (see Appendix D and Appendix E).

7.7 Follow-up visit

For participants subject to HIV testing, test results and appropriate counseling will be provided at a second clinic visit. This visit is not required for participants who have been diagnosed with HIV infection prior to enrolling in this sub-study.

7.8 Post-study contact

Following completion of the sub-study, the CRS will contact the participant and provide information about sub-study results and any new understanding of Phambili that has been gained. CRSs should document in the participant study record completion of (and unsuccessful attempts to complete) this post-study contact.

[a] For placebo recipients who have enrolled in another HVTN clinical trial that includes HIV testing and who have not yet completed their scheduled clinic visits, results from their most recent HIV tests in that trial may be recorded for HVTN 503-S. Additional HIV testing in HVTN 503-S is not required for these individuals.

7.9 Visit windows

Visit windows are defined in HVTN 503-S Study Specific Procedures.

7.10 VISP registry consent form

Experimental HIV vaccines may induce antibody production to HIV antigens, producing reactive results on commercially available HIV test kits. This is called “vaccine-induced seropositivity” (VISP). In order to provide post-study HIV testing to distinguish between VISP and HIV infection, and to mitigate potential social harms resulting from VISP in HIV vaccine recipients who are not infected with HIV, the HVTN has created a VISP registry. The registry allows trained staff to verify that an individual has received an HIV vaccine, and therefore has the potential for VISP. Information in the VISP registry will not be used for research. Rather, the registry exists to support provision of post-study testing and counseling services to HIV vaccine recipients.

The VISP registry consent form describes the purpose of the VISP registry, the participant information to be included in the registry, confidentiality protections, and risks and benefits associated with inclusion in the registry. The VISP registry consent form is contained in Appendix C.

The VISP Registry consent form will be presented to all HVTN 503-S participants.

8 Laboratory

The HVTN Site Lab Reference Manual provides guidelines for phlebotomy, specimen labeling, whole blood processing, and HIV diagnostic testing. Tube types and volumes for blood collection are specified in Appendix D and Appendix E.

8.1 Exploratory studies

Samples may be used for testing and research related to furthering the understanding of HIV immunology or vaccines. In addition, cryopreserved samples may be used to perform additional assays to support standardization and validation of existing or newly developed methods.

8.2 Other use of stored specimens

The HVTN stores specimens from all study participants indefinitely, unless a participant requests that specimens be destroyed or if required by Institutional Review Board (IRB)/Ethics Committee (EC), or Regulatory Entity (RE).

Other use of specimens is defined as studies not described in the protocol.

This research may relate to HIV, vaccines, the immune system, and other diseases. This could include limited genetic testing and, potentially, genome-wide studies. This research is done only to the extent authorized in each study site’s informed consent form, or as otherwise authorized under applicable law. Other testing on specimens will occur only after review and approval by the HVTN, the IRB/EC of the researcher requesting the specimens, and the CRS’s IRBs/ECs if required.

The protocol sample informed consent form (see Appendix B) is written so that the participant either explicitly allows or does not allow their samples to be used in other research when they sign the form. Participants who initially agree to other use of their samples may rescind their approval at any time; such participants will remain in this study and their samples will only be used for the studies described in this protocol. If a participant decides against allowing other research using his or her samples, or at any time rescinds prior approval for such other use, the study site investigator or designee must notify HVTN Regulatory Affairs in writing. In either case, HVTN Regulatory Affairs directs the HVTN Lab Program not to use samples from these participants for such other uses.

CRSs must notify HVTN Regulatory Affairs if institutional or local governmental requirements pose a conflict with or impose restrictions on other use of specimens.

8.3 Biohazard containment

As the transmission of HIV and other blood-borne pathogens can occur through contact with contaminated needles, blood, and blood products, appropriate precautions will be employed by all personnel in the drawing of blood and shipping and handling of all

specimens for this study, as currently recommended by the CDC and the NIH or other applicable agencies.

All dangerous goods materials, including Biological Substances, Category A or Category B, must be transported according to instructions detailed in the International Air Transport Association Dangerous Goods Regulations.

9 Protocol conduct

This protocol and all actions and activities connected with it will be conducted in compliance with the principles of Good Clinical Practice (GCP) (ICHe6), and according to DAIDS and HVTN policies and procedures as specified in the HVTN Manual of Operations, DAIDS Clinical Research Policies and Standard Procedures Documents including procedures for the following:

- Protocol registration, activation, and implementation;
- Informed consent and enrollment;
- Study participant reimbursement;
- Data collection, documentation, transfer, and storage;
- Participant confidentiality;
- Study follow-up and close-out;
- Quality control;
- Risk reduction counseling;
- Specimen collection, processing, and analysis;
- Destruction of specimens.

Any policies or procedures that vary from DAIDS and HVTN standards or require additional instructions will be described in the HVTN 503-S Study Specific Procedures.

As no investigational product will be administered as part of this study, no specific additional tests for post-procedure clinical monitoring or for AE or EAE grading and reporting will be implemented.

10 Document references (other than literature citations)

Other documents referred to in this protocol, and containing information relevant to the conduct of this study, include:

- Current CDC Guidelines. Revised Recommendations for HIV Testing of Adults, Adolescents, and Pregnant Women in Health-Care Settings. Available at http://www.cdc.gov/mmwr/PDF/rr/rr5514.pdf.
- Division of AIDS (DAIDS) Clinical Research Policies and Standard Procedures Documents. Available at http://www3.niaid.nih.gov/research/resources/DAIDSClinRsrch/
- Division of AIDS Protocol Registration Manual. Available at http://www.niaid.nih.gov/LabsAndResources/resources/DAIDSClinRsrch/Documents/prmanual.pdf
- HVTN 503-S Special Instructions. Accessible through the HVTN protocol-specific website.
- HVTN 503-S Study Specific Procedures. Accessible through the HVTN protocol-specific website.
- HVTN Site Lab Reference Manual. Accessible through the HVTN website.
- HVTN Manual of Operations. Accessible through the HVTN website.
- Dangerous Goods Regulations (updated annually), International Air Transport Association. Available for purchase at http://www.iata.org/ps/publications/dgr/Pages/index.aspx.
- HVTN algorithm for diagnosis of HIV infections. Part of the HVTN Site Lab Reference Manual (see above).
- International Conference on Harmonisation (ICH) E6 (R1), Guideline for Good Clinical Practice: Section 4.8, Informed consent of trial subjects. Available at http://www.ich.org/fileadmin/Public_Web_Site/ICH_Products/Guidelines/Efficacy/E6_R1/Step4/E6_R1__Guideline.pdf
- Participants' Bill of Rights and Responsibilities. Accessible through the HVTN website.
- Requirements for Source Documentation in DAIDS Funded and/or Sponsored Clinical Trials. Available at https://phacs.nichdclinicalstudies.org/publicDocs/DAIDS_SourceDocPolicy.pdf

See Section 12 for literature cited in the background and statistics sections of this protocol.

11 Acronyms and abbreviations

Ad5	adenovirus serotype 5
CAB	Community Advisory Board
CDC	US Centers for Disease Control and Prevention
CRS*	clinical research site
DAIDS	Division of AIDS (US NIH)
DHHS	US Department of Health and Human Services
EC	Ethics Committee
FHCRC	Fred Hutchinson Cancer Research Center
GCP	Good Clinical Practice
HIV	human immunodeficiency virus
HVTN	HIV Vaccine Trials Network
ICH	International Conference on Harmonisation
IND	Investigational New Drug
IRB	Institutional Review Board
NIAID	National Institute of Allergy and Infectious Diseases (US NIH)
NIH	US National Institutes of Health
SCHARP	Statistical Center for HIV/AIDS Research and Prevention
SDMC	statistical and data management center
VISP	vaccine-induced seropositivity

* CRSs were formerly referred to as HIV Vaccine Trial Units (HVTUs). Conversion to use of the term CRS is in process, and some HVTN documents may still refer to HVTUs.

12 Literature cited

1. UNAIDS. Ethical considerations in biomedical HIV prevention trials. **2007**.

2. The National Commission for the Protection of Human Subjects of Biomedical and Behavioral Research. The Belmont Report: Ethical Principles and Guidelines for the Protection of Human Subjects of Research. **1979**.

3. Council for International Organizations of Medical Sciences (CIOMS). International ethical guidelines for biomedical research involving human subjects. Bull Med Ethics **2002**;17-23.

4. Buchbinder SP, Mehrotra DV, Duerr A, Fitzgerald DW, Mogg R, Li D, Gilbert PB, Lama JR, Marmor M, Del Rio C, McElrath MJ, Casimiro DR, Gottesdiener KM, Chodakewitz JA, Corey L, Robertson MN. Efficacy assessment of a cell-mediated immunity HIV-1 vaccine (the Step Study): a double-blind, randomised, placebo-controlled, test-of-concept trial. Lancet **2008**;372:1881-93.

5. Duerr A, Huang Y, Buchbinder S, Coombs RW, Sanchez J, Del RC, Casapia M, Santiago S, Gilbert P, Corey L, Robertson MN. Extended Follow-up Confirms Early Vaccine-Enhanced Risk of HIV Acquisition and Demonstrates Waning Effect Over Time Among Participants in a Randomized Trial of Recombinant Adenovirus HIV Vaccine (Step Study). J Infect Dis **2012**;206:258-66.

6. Gray GE, Allen M, Moodie Z, Churchyard G, Bekker LG, Nchabeleng M, Mlisana K, Metch B, de BG, Latka MH, Roux S, Mathebula M, Naicker N, Ducar C, Carter DK, Puren A, Eaton N, McElrath MJ, Robertson M, Corey L, Kublin JG. Safety and efficacy of the HVTN 503/Phambili study of a clade-B-based HIV-1 vaccine in South Africa: a double-blind, randomised, placebo-controlled test-of-concept phase 2b study. Lancet Infect Dis **2011**;11:507-15.

Appendix A Sample informed consent form

Title: HIV testing and behavioral risk assessment for former HVTN 503 (Phambili) participants

HVTN protocol number: HVTN 503-S

Site: [Insert site name]

Please read this consent form or ask someone to read it to you. If you decide to join the study, we will ask you to sign or make your mark on this form. We will offer you a copy to keep. You can also ask us questions about the study.

You are being asked to join this study because you were enrolled in the Phambili study. The Phambili study tested a vaccine called MRKAd5 HIV-1 gag/pol/nef. We now have information from the completed study. We see that more people who got the study vaccine got HIV infection than people who got the placebo. There could be many reasons for this:

- Many people left the study early. This makes it hard to know for sure if there were more infections in the group that received vaccine.
- It may be that sexual practices differed between people who got the vaccine and people who got the placebo.
- There may be some other reason that we don't understand yet.

We need to understand more about the results of Phambili. That is why we are asking you to join this research study. As many as 700 former Phambili participants will be ask to enroll in this study.

1. **We are doing this study to learn more about:**

- How many people in Phambili have become infected with HIV
- How people's sexual behaviors may have changed over time
- How people's bodies responded to different parts of the study vaccine

2. **It is completely up to you whether or not to join the study.**

If it helps, talk to people you trust, such as your doctor, friends, or family. If you decide not to join this study, your other care at this clinic and the benefits or rights you would normally have will not be affected.

3. **If you join the study, you will come to the clinic for scheduled visits about 2 times over 2 weeks.**

Visits can last from [#] to [#] hours.

At the first visit, we will:

- Conduct a physical examination. We will:
    - Check your height, weight, temperature, and blood pressure;
    - Look in your mouth and throat;
    - Listen to your heart and lungs;
    - Feel your abdomen;
    - Do a genital exam (men only);
- Ask if you remember whether you got the vaccine or the placebo. We will make sure we gave you the correct information;
- Ask you about any HIV testing you have gotten since you were in Phambili;
- Ask you about your sexual practices and sexual partners;
- Counsel you on HIV prevention;
- Collect blood samples to:
    - Test for HIV, and
    - Store for possible later testing.

We will collect about 55 mL (a little less than one quarter of a cup) of blood for this testing.

If you have already been diagnosed with HIV, we will ask you where and when you received your HIV diagnosis.

If you got the vaccine in Phambili and were diagnosed with HIV at a Phambili clinic, we will not collect blood for another HIV test. We will ask that clinic for a copy of your test results. If you tested HIV-positive at a clinic not associated with Phambili, we will do another HIV test. We want to be sure you have HIV.

If you got the placebo in Phambili and have been diagnosed with HIV, we will not collect blood for another HIV test. If you got the placebo in Phambili and are still attending scheduled clinic visits in another HVTN vaccine trial, we will record your most recent HIV test result from that trial. You do not need to have another HIV test as part of this study.

If we do an HIV test, we will ask you to come back for a second visit. We will give you the test results at that visit.

We may ask you to return to the clinic if we need to collect more samples or redo any of the tests.

Most of your samples will be stored and tested in South Africa, but some may be shipped to the US for some tests, including limited genetic testing.

If you are a man and have been circumcised, we will ask you when and where your circumcision took place.

After this study is over, we will contact you to tell you what we have learned. If you want, you can come to the clinic to talk about this.

4. **We will give you [Site: Insert compensation] for each study visit you complete.**

This amount is to cover the costs of [Site: Insert text]

You do not have to pay anything to be in this study.

5. **We will counsel you on HIV prevention.**

We will ask you personal questions about your sexual practices. We will talk with you about ways of lowering your risk of getting HIV. If you are infected with HIV, we will talk with you about ways to avoid giving the virus to someone else and help you access care. We will help you develop a risk reduction plan. Some topics we may discuss include:

- What you think causes risky behavior for you.
- Methods to avoid getting HIV or giving it to someone else.

These may include regular HIV testing, getting circumcised if you are a man, not having sex, using condoms, or other behaviour changes, such as cutting down on alcohol or knowing your partner’s HIV status.

6. **We will do our best to protect your private information.**

Your study records and samples will be kept in a secure location. We will label all of your samples and most of your records with a code number, not your name or other personal information. We will not share your name with the lab that does the tests on your samples, or with anyone else who does not need to know your name.

Clinic staff will have access to your study records. Your records may also be reviewed by groups who watch over this study to see that we are protecting your rights, keeping you safe, and following the study plan. These groups include:

- [Insert name of local IRB/EC] ,
- The South Africa Medicines Control Council,
- The US National Institutes of Health and people who work for them,
- The US Food and Drug Administration,
- Merck & Co., Inc. and people who work for them,
- The HIV Vaccine Trials Network (HVTN) and people who work for them, and

- The US Office for Human Research Protections.

All reviewers will take steps to keep your records private.

We cannot guarantee absolute privacy. At this clinic, we have to report the following information:

Site: Include any public health or legal reporting requirements. Bulleted examples should include all appropriate cases (reportable communicable disease, risk of harm to self or others, etc.).

- [Item 1]
- [Item 2]
- [Item 3]

The results of this study may be published. No publication will use your name or identify you personally.

We may share information from the study with other researchers. We will not share your name or personal information.

When the study is done, we may share the information from the study with others so they can see it and use it. We will not share any information that will let someone identify you.

Risks

7. There may be risks to being in this study.

In this study, we will take some blood samples. This can cause bruising, pain, fainting, soreness, redness, swelling, itching, muscle damage, and (rarely) infection where the needle was inserted.

You may feel embarrassed when we ask about your sexual practices or about drug or alcohol use. Parts of the physical examination may make you feel embarrassed or anxious. Also, waiting for your HIV test results could make you feel anxious. You could feel worried if your test results show that you are infected with HIV. If you feel embarrassed or anxious, please tell us and we will try to help you.

Although the risk is very small, it is possible that your personal information could be given to someone who should not have it. If that happened, you could face discrimination, stress, and embarrassment. We can tell you more about how we will protect your personal information if you would like it.

Benefits

8. The study may not benefit you.

The counseling that you get as part of the study may help you avoid getting HIV. The lab tests and physical exams that you get while in this study might detect health problems you don't yet know about.

Your rights and responsibilities

9. If you join the study, you have rights and responsibilities.

You have many rights that we will respect. You also have responsibilities. We list these in the Participant's Bill of Rights and Responsibilities. We will give you a copy of it.

Leaving the study

10. Tell us if you decide to leave the study.

You are free to leave the study at any time and for any reason. Your care at this clinic and your legal rights will not be affected, but it is important for you to let us know.

Injuries

11. If you get sick or injured during the study, contact us immediately.

Your health is important to us. We will help you get the medical care you need.

If someone gets sick or injured in an HVTN study, the HVTN decides whether the injury is probably related to the study procedures. If the HVTN decides it was more likely due to the study procedures than any other cause, then the HVTN will use their funds to pay for treatment. The HVTN has limited funds to cover the cost of medical treatment for injuries caused by study procedures.

No matter what, you still have the right to use the court system to address payment for your injuries if you are not satisfied.

Some injuries are not physical. For example, someone might be harmed psychologically or emotionally by being in an HIV study. Or they might lose wages from injuries because they could not go to work. No funds have been set aside to pay for nonphysical injuries, even if they are related to participation in the study.

Questions

12. If you have questions or problems at any time during your participation in this study, use the following important contacts.

If you have questions about this study, contact
[name and telephone number of the investigator or other study staff].

If you have any symptoms that you think may be related to this study, contact [name and telephone number of the investigator or other study staff].

If you have questions about your rights as a research participant, or problems or concerns about how you are being treated in this study, contact [name/title/phone of person on IRB or other appropriate organization].

If you want to leave this study, contact [name and telephone number of the investigator or other study staff].

If you do not feel your questions or concerns have been answered to your satisfaction after talking to the contacts above, you should write to the South African Medicines Control Council (MCC) at: The Registrar, SA Medicines Control Council, Department of Health, Private Bag X828, Pretoria 0001

Your permissions and signature

13. If you agree to join this study, you will need to sign or make your mark below. Before you sign or make your mark on this consent form, make sure of the following:

- You have read this consent form, or someone has read it to you.
- You feel that you understand what the study is about and what will happen to you if you join. You understand what the possible risks and benefits are.
- You have had your questions answered and know that you can ask more.
- You agree to join this study.

You will not be giving up any of your rights by signing this consent form.

Participant's name (print)	Participant's signature or mark	Date	Time
Clinic staff conducting consent discussion (print)	Clinic staff signature	Date	Time

For participants who are unable to read or write, a witness should complete the signature block below:

Witness's name (print)	Witness's signature	Date	Time

*Witness is impartial and was present for the consent process.

Appendix B Sample consent form for use of samples and information in other studies

Title: HIV testing and behavioral risk assessment for former HVTN 503 (Phambili) participants

HVTN protocol number: HVTN 503-S

Site: [Insert site name]

When samples are no longer needed for this study, the HVTN wants to keep them for use in other studies. We will call these “extra samples.”

This form gives you information so you can decide if you want your extra samples and information used in other studies. You will mark your decision at the end of the form. If you have any questions, please ask.

1. Do I have to agree?

No. You are free to say yes or no, or to change your mind after you sign this form. Your decision will not affect your being in this study or have any negative consequences here.

2. Where are the samples stored?

Extra samples are stored in a secure central place called a repository. *[Site: insert specific information if your regulatory authority requires it.]* The central repositories for the HVTN are located in the United States and South Africa.

Depending on what studies are needed, some of your samples may be shipped to laboratories in the United States for testing.

3. How long will the samples be stored?

There is no limit on how long your extra samples will be stored. *[Site: insert limits if your regulatory authority imposes them.]*

4. Will I be paid for the use of my samples?

No. Also, a researcher may make a new scientific discovery or product based on the use of your samples. If this happens, there is no plan to share any money with you. The researcher is not likely to ever know who you are.

5. Will I benefit from allowing my samples to be used in other studies?

Probably not. Results from these other studies are not given to you, this clinic, or your doctor. They are not needed for your medical care. They are not part of your medical record. The studies are only being done for research purposes.

6. Will the HVTN sell my samples and information?

No, but the HVTN may share your samples with other researchers.

7. How do other researchers get my samples and information?

When a researcher wants to use your samples and/or information, their research plan must be approved by the HVTN. Also, the researcher's institutional review board (IRB) or ethics committee (EC) will review their plan. *[Site: insert review by your institution's IRB/EC, if applicable.]* IRBs/ECs protect the rights and well-being of people in research. The HVTN keeps track of your decision about how your samples and information can be used.

8. What information is shared with other researchers?

We will not share any information that would make it easy for anyone to identify you. However, some information that we share may be personal, such as your race, ethnicity, gender, health information from the study, and HIV status. We may share information about the study product you received and how your body responded to the study product.

9. How is my privacy protected?

Your samples are labeled with a code instead of your name. We will not tell the researchers who you are.

10. What kind of studies might be done with my extra samples and information?

The studies will be related to HIV, vaccines, the immune system and other diseases. Researchers will need to ask your permission for other types of research not described in this informed consent. Tell us below if you want researchers to contact you about using your extra samples for studies that are not mentioned in this consent.

The studies will probably include genetic testing. Your genes are passed to you from your birth parents. Genes are the basic "instruction book" for the cells that make up our bodies. The differences in people's genes can help explain why some people get a disease while others do not.

Researchers will only look at the genes that may be related to the immune system and diseases. They will not look at all of your genes (your genome). We call this "limited genetic testing". For example, researchers may look at genes that affect how you fight infection. <u>If you agree to have your samples and information used for other studies, you are also agreeing to this kind of limited genetic testing.</u>

11. What are the risks of limited genetic testing?

The genetic testing could show you may be at risk for certain diseases or that you are or are not related to someone. If others found out, it could lead to discrimination or other problems. However, it is almost impossible for you or others to know your test results from extra samples. Remember:

- The results are not given to you, this clinic, or your doctor.
- The results are not linked to your name.
- The researchers who use your samples and information have agreed never to find out who you are.

- The results are not a part of your medical record.

Someone could try to find out who you are. For this to happen, that person would have to get into another database that links your study information and your name. The risk of this is very small.

12. Who will have access to my information in studies using my extra samples?

Some people will be able to see the research records from any new study that uses your extra samples and information. Remember that your name will not be part of the information.

People who may see your information are:

- Government agencies that fund or monitor the research using your samples or information
- The researcher's Institutional Review Board or Ethics Committee
- The people who work with the researcher

All reviewers will take steps to keep your records private.

The results of any new studies that use your extra samples or information may be published. No publication will use your name or identify you personally.

Questions

13. If you have questions or problems about allowing your samples and information to be used in other studies, use the following important contacts.

If you have questions about the use of your samples or information or if you want to change your mind about their use, contact
[name and telephone number of the investigator or other study staff].

If you think you may have been harmed because of studies using your samples or information, contact [name and telephone number of the investigator or other study staff].

If you have questions about your rights as a research participant, contact
[name/title/phone of person on IRB or other appropriate organization].

14. Please write your initials or make your mark in the box next to the option you choose.

☐ I agree to allow my extra samples and information to be used for other studies related to HIV, vaccines, the immune system and other diseases. This may include limited genetic testing.

OR

☐ I do not agree to allow my extra samples and information to be used in other studies.

☐ I agree to be contacted in the future for other types of research not described in this informed consent.

Participant's name (print)	Participant's signature or mark	Date	Time
Clinic staff conducting consent discussion (print)	Clinic staff signature	Date	Time

For participants who are unable to read or write, a witness should complete the signature block below:

Witness's name (print)	Witness's signature	Date	Time

*Witness is impartial and was present for the consent process.

Appendix C HVTN VISP registry consent

The HIV Vaccine Trials Network (HVTN) would like your permission to enter your name and link it to information about you in a computer registry (the “VISP registry”). By having your name and vaccine study information in the VISP registry, trained staff can quickly help you if you have problems with VISP.

About VISP

The body makes antibodies to prevent infection. Most vaccines cause the body to make antibodies as a way of preventing infection. Your body may make antibodies to HIV because you received an HIV vaccine. Some HIV test results could come back positive even if you are not infected with HIV. This is called a VISP (vaccine-induced seropositive) test result. We do not know who will have VISP test results or how long these test results may last.

People with VISP test results need specific HIV tests. These tests can tell whether a test result is VISP or a real HIV infection. These people may need help explaining their VISP situation if someone outside the study wants to test them for HIV. VISP test results may cause problems in several areas like insurance, job applications, the military, prison, visa applications, emigration/immigration, and blood and tissue donation.

We are asking you for your permission to enter your name in the registry now in case you have VISP test results later. The registry will not be used for any other purpose.

What are the benefits of the registry?

Your study site will help you with problems related to VISP test results. The site staff will need to verify your study participation and if you received an HIV vaccine. The registry gives the site staff quick access to this information.

If you choose not to have your name entered in the registry, site staff still will do their best to help you. However, it will take longer to get that information. If your study site is no longer doing HIV vaccine studies, your records may be stored securely off site. It is possible your records may not be found.

What information does the registry contain and how is it protected?

The registry contains the following information:

- Your participant ID (the code used for you instead of your name at your study site)
- The study network and study you were in
- The site where you began the study
- The date you began the study

- Your date of birth or age
- If you received an HIV vaccine that may cause you to test VISP

We are asking for your permission to enter your name into the registry and link it to the information above.

The registry will NOT contain:

- Your HIV test results
- Your phone number or any other way to contact you

Any other personal information that you discuss with the site staff will be kept separate from the registry. We will keep your name in the registry until you tell us you want it removed.

All people who work with your registry information sign agreements to keep the information confidential.

The registry is a secured computer database. It can only be accessed with a password.

What are the risks?

The only risk to having your name entered and linked to the other pieces of information in the registry is that someone who is not authorized might see your information. The risk of this happening is low because of the security protections in place. However, we cannot guarantee this will never happen.

What if I have more questions about the registry?

Please talk to your study site if you have any questions about the registry now or in the future.

If I agree now, can I change my mind later?

Yes. You can contact your study site to tell them that you would like your name to be deleted from the registry. Your decision will not affect your participation in the main HIV vaccine study.

By signing this form, you do not give up any legal rights.

Please write your initials or make your mark in the box next to the option you choose.

☐ I **AGREE** to allow my name to be entered and linked to the information in the HVTN VISP registry.

☐ I **DO NOT AGREE** to allow my name to be entered and linked to the information in the HVTN VISP registry.

Please sign or make your mark below.

Participant's name (print)	Participant's signature or mark	Date	Time
Study staff conducting consent discussion (print)	Study staff signature	Date	Time

For participants who are unable to read or write, a witness should complete the signature block below:

Witness's name (print)#	Witness's signature	Date	Time

Witness is impartial and was present for the consent process.

Appendix D Laboratory procedures (placebo recipients)

				Visit:	1	2	Total
				Day:	D0	D14	
				Month	M0	M0.5	
**Procedure**	**Ship to[1]**	**Assay location**	**Tube Type[2]**	**Tube size (vol. capacity)[2]**			
BLOOD COLLECTION							
Screening/Diagnostic Assay							
Screening HIV Test[3]	Local lab	Local lab	SST	10mL	10	—	**10**
Storage							
PBMC	CSR	—	ACD	8.5mL	34	—	**34**
Serum	CSR	—	SST	8.5mL	8.5	—	**8.5**
**Visit total**					**53**	**0**	**53**
**56-Day total**					**53**	**53**	

[1] CSR = central specimen repository
[2] Local labs may assign appropriate alternative tube types for locally performed tests.
[3] Screening HIV test not required for placebo recipients previously diagnosed with HIV infection. For Phambili placebo recipients who have enrolled in another HVTN clinical trial that includes HIV testing and who have not yet completed scheduled clinic visits in that trial, results from their most recent HIV tests in that trial may be recorded for HVTN 503-S. Additional HIV testing in HVTN 503-S is not required for these individuals.

Appendix E Laboratory procedures (vaccinees)

				Visit:	1	2	
				Day:	D0	D14	
				Month	M0	M0.5	
**Procedure**	**Ship to[1,2]**	**Assay location[2]**	**Tube Type**	**Tube size (vol. capacity)**			**Total**
BLOOD COLLECTION							
Screening/Diagnostic Assay							
HIV diagnostic algorithm[3]	SV/SMDU-NICD	SV/SMDU-NICD	EDTA	10mL	10	—	**10**
Storage							
PBMC	CSR	—	ACD	8.5mL	34	—	**34**
Serum	CSR	—	SST	8.5mL	8.5	—	**8.5**
**Visit total**					**53**	**0**	**53**
**56-Day total**					**53**	**53**	

[1] CSR = central specimen repository

[2] HVTN Laboratory Program includes laboratories at SV/SMDU-NICD. SV/SMDU-NICD = Serology-Virology & Special Molecular Diagnostic Unit, National Institute for Communicable Diseases (Johannesburg, South Africa)

[3] HIV testing not required for vaccine recipients previously diagnosed with HIV at a Phambili CRS.

Appendix F Procedures at HVTN CRS

	01	02[a]	Post[b]
Day:	D0	D14	
Month:	M0	0.5	
**Study procedures[c]**			
Signed protocol consent	X	—	—
Obtain recall of treatment assignment, confirm treatment assignment	X	—	—
Complete physical examination[d]	X	—	—
Risk reduction counseling	X	—	—
Behavioral risk questionnaire	X	—	—
HIV testing and counseling[e]	X	—	—
Provide HIV test results (include post-test counseling)[a]	—	X	—
Provide updated Phambili and sub-study results	—	—	X

[a] Not required for participants for whom HIV tests are not performed in this sub-study (see Section 7.4).
[b] At participant's discretion, this may be a clinic visit or contact via telephone or other appropriate means (see Section 7.8).
[c] For specimen collection requirements, see Appendix D and Appendix E.
[d] For male participants, includes assessment of circumcision status.
[e] HIV test not required for placebo recipients previously diagnosed with HIV or for vaccinees diagnosed with HIV at a Phambili CRS (see Section 7.4).
